# Evaluation of dermatologic diagnostic ability on skin of colour in preclinical medical students

**DOI:** 10.1002/ski2.425

**Published:** 2024-09-03

**Authors:** Adina Greene, Tara Ghalambor, Scott Penner, Chase Irwin, K. Taraszka Hastings

**Affiliations:** ^1^ University of Arizona College of Medicine‐ Phoenix Phoenix Arizona USA; ^2^ Mayo Clinic Department of Dermatology Scottsdale Arizona USA; ^3^ Phoenix Veterans Affairs Health Care System Phoenix Arizona USA

## Abstract

**Background:**

Various studies have revealed that there is a disproportionately low representation of skin of colour (SOC) in medical school dermatologic curriculum and board study resources.

**Methods:**

First‐year and second‐year medical students were emailed an 18‐question survey regarding (1) identifying correct diagnoses of dermatologic conditions on either White skin or SOC and (2) their confidence in identifying dermatologic conditions on SOC.

**Results:**

15% of the images of dermatologic conditions included in the institutional preclinical curriculum show images of patients with SOC. Regarding overall scores for diagnosing dermatologic diseases, students performed similarly on both the White image survey (61.73%) and SOC image survey (66.20%) with no statistically significant differences between surveys (*p* = 0.14). Second‐year medical students performed better than first‐year medical students overall (*p* = 0.01) and on White skin image survey scores (*p* = 0.02) but not on people of colour image survey scores (*p* = 0.09). Students largely agreed that they were more comfortable identifying dermatologic diagnoses on White skin and that their school could benefit from increased SOC dermatological resources.

**Conclusion:**

The overall low scores for the diagnosis of common skin conditions on both the White image and SOC image survey by first‐ and second‐year students are not surprising given the results of a prior study and support the need for re‐exposure to dermatology presentations in all skin types during the preclinical curriculum. The low scores support the need for changes in the pre‐clinical dermatology curriculum to improve diagnostic ability. Second‐year students performed better at diagnosing dermatologic conditions overall and on White skin compared to SOC at the end of their didactic years, possibly due to an underrepresentation of SOC images in institutional and outside educational resources. Attitudes towards school‐specific dermatologic SOC education demonstrated a clear desire amongst students for more exposure to dermatologic conditions in various skin colours throughout the curriculum.

**What is already known?**

- Various studies have revealed that there is a disproportionately low representation of skin of colour (SOC) in medical school dermatologic curricula and board study resources.

**What does this study add?**

- This analysis helps evaluate which areas students continue to struggle diagnosing common dermatological diseases on SOC and can serve as a foundation for medical schools nationally to evaluate their SOC curriculum in preclinical years.

## 1 INTRODUCTION

Dermatologic health disparities have been well documented in people of colour (POC).^1^ While these disparities are multifactorial, one contributing factor is dermatologic training. Various studies have revealed a disproportionately low representation of SOC in medical education in the United States.^1^, ^2^ Commonly used third‐party board preparatory resources such as Pathoma, Amboss and Boards and Beyond report a low proportion of SOC images with a total of 21.4%, 7.2% and 4.8% SOC images, respectively.^3^, ^4^ This lack of representation of SOC begins at the medical school level and continues throughout residency, fellowship and continuing education.^2^

While the University of Arizona College of Medicine‐Phoenix (UACOMP) encouraged faculty to increase the representation of skin diseases on SOC in the images used during the preclinical curriculum, the representation of SOC throughout the first‐ and second‐year curriculum has not been evaluated. This study assessed the representation of SOC throughout the first‐ and second‐year curriculum, the ability of medical students to diagnose common skin conditions on SOC versus White skin and confidence levels with exposure to dermatologic conditions on all skin types.

## 2 METHODS

This study was approved by the University of Arizona IRB. First‐ and second‐year medical students at UACOMP were recruited through email and presented with a quiz after demographic questions consisting of 15 multiple‐choice questions assessing dermatological diagnostic ability, and 3 Likert‐Scale questions asking participants to rate their confidence level of identifying dermatologic conditions on SOC and the diversity of dermatologic resources. Inclusion criteria for selected dermatologic diagnoses included all diagnoses taught in the first 6 months of UACOMP curriculum including Lyme disease, acne, atopic dermatitis, psoriasis, varicella, folliculitis, tinea versicolour, melanoma, hypertrophic scar, urticaria, impetigo, molluscum contagiosum, café au lait maculae, neurofibroma and port wine stain. Surveys were created using images from the American Academy of Dermatology, CDC, DermNet, Skinsight and Skin Deep.

### 2.1 Statistical methods

Descriptive statistics were stratified by the survey administered and were reported as frequencies and proportions. Chi‐square tests were used to evaluate differences across strata. For our primary outcome, test scores were calculated for the White image survey and SOC image survey for the full cohort. We performed sub‐group analyses by further stratifying our cohort by racial identity (POC vs. White students) and year in school (MS1 vs. MS2). We reported the mean, standard deviation and percent correct for each strata. Either two‐sample independent *t*‐tests or Mann–Whitney U tests with Benjamini & Hochberg's adjustment for multiple comparisons were used to evaluate differences in overall scores and across sub‐groups (Table 1).

**TABLE 1:** Overall test score results; white skin images versus skin of colour (SOC) images (*n* = 91).

	White image survey (*n* = 46)	SOC image survey (*n* = 45)	*p*‐value^b^
Overall score^b^, mean (SD) [% correct]	9.26 (1.91) [61.73]	9.93 (2.39) [66.20]	0.28
POC versus white students			
	POC (*n* = 25)	White (*n* = 66)	
Overall score, mean (SD) [% correct]	9.48 (1.85) [63.20]	9.64 (2.30) [64.27]	0.76
White image survey	POC (*n* = 14)	White (*n* = 32)	
White image survey score, mean (SD) [% correct]	8.71 (1.54) [58.07]	9.50 (2.03) [63.33]	0.60
SOC image survey	POC (*n* = 11)	White (*n* = 34)	
SOC image survey score, mean (SD) [% correct]	10.45 (1.81) [69.67]	9.76 (2.55) [65.07]	0.62
MS1 versus MS2 students			
	MS1 (*n* = 49)	MS2 (*n* = 42)	
Overall score, mean (SD) [% correct]	9.00 (2.38) [60.00]	10.29 (1.69) [68.60]	0.01^a^
White image survey	MS1 (*n* = 25)	MS2 (*n* = 21)	
White image survey score, mean (SD) [% correct]	8.64 (2.10) [57.60]	10.00 (1.38) [66.67]	0.02^a^
SOC image survey	MS1 (*n* = 24)	MS2 (*n* = 21)	
SOC image survey score, mean (SD) [% correct]	9.38 (2.63) [62.50]	10.57 (1.94) [70.47]	0.09

^a^ Indicates statistically significant result for alpha level = 0.05.

^b^ Two‐sample independent *t*‐test or Mann–Whitney *U* test with Benjamini & Hochberg's adjustment for multiple comparisons.

Responses to all Likert scale questions were scored as strongly disagree = 1, disagree = 2, neutral = 3, agree = 4 and strongly agree = 5. We reported the mean and standard deviation of the full cohort for each question. All analyses were performed in SAS version 9.4 (SAS Institute, Cary, NC).

## 3 RESULTS

Overall, 91 medical students completed the surveys out of 109 recruited students (response rate = 83.5%). In total, 46 students completed the White image survey with 14% identifying as a POC and 54% being first‐year students, and 45 students completed SOC image survey with 11% identifying as a POC and 53% being first‐year students. There were no significant differences in the year in medical school (*p* = 0.93) or percentage of POC (*p* = 0.52) between the cohorts taking the White and SOC image surveys.

Quantification of the pre‐clinical lectures at UACOMP revealed that 15% of the total images were SOC. Regarding overall scores for diagnosing dermatologic diseases, students performed similarly on both the White and SOC image surveys (*p* = 0.28). When conducting sub‐group analyses, there were no statistically significant differences on White image survey versus SOC image survey for those who identify as a POC versus those who do not (Table 1). Students were more likely to identify psoriasis (*p* = 0.004), varicella zoster (*p* = 0.004) and melanoma (*p* = 0.01) on White skin and neurofibroma (*p* = 0.004) and tinea versicolour (*p* = 0.004) on SOC. Second‐year medical students scored better than first‐year medical students overall (*p* = 0.01) and on the White image survey scores (*p* = 0.02) but not on POC image survey scores (*p* = 0.09) (Table 1).

Students largely agreed that they were more comfortable identifying dermatologic diagnoses on White skin (Likert scale = 4.05) and that more opportunities to identify common skin lesions on SOC would enhance their medical education (Likert scale = 4.56).

## 4 DISCUSSION

We investigated the diagnostic abilities of medical students with regard to the presentation of dermatologic conditions in different skin colours. Students performed similarly on both White and SOC image surveys. The overall low scores are not surprising given the results of a prior study at Tulane University where preclinical students had an average of 47.3% on a similar multiple‐choice dermatology quiz.^5^ Similar to prior studies, students are more likely to correctly diagnose skin conditions presenting with erythema, such as psoriasis, on White skin and skin conditions presenting with a change in pigmentation, such as tinea versicolour, on SOC.^5^, ^6^, ^7^ Students were less likely to correctly diagnose melanoma on SOC. Since melanoma tends to be diagnosed at later stages in POC contributing to increased morbidity and mortality, it is important to improve the pre‐clinical curriculum on the presentation of melanoma on SOC.^8^

Subgroup analysis showed that second‐year medical students outperformed first‐year medical students on the overall score for the White image survey but not on the SOC image survey. The survey was distributed to second‐year students after a 6‐week dedicated study for Step 1, and thus they may have had a better knowledge foundation. One hypothesis for this difference is that there is a lower proportion of SOC images in widely used third‐party Step 1 preparatory materials used primarily by second‐year students.^4^, ^9^, ^10^ However, these results should be interpreted cautiously as the numerical improvement may not demonstrate clinically significant increase in diagnostic ability.

Analysis of the attitudes towards school‐specific dermatological SOC education demonstrated a clear desire amongst students for more exposure to dermatologic conditions in various skin colours. We hope that continued publications highlighting these concerns, in addition to work done by advocacy groups such as the SOC Society, will push medical schools, resource companies and the National Board of Medical Examiners to increase the diversity of their curriculum. While it is important for medical schools to implement these changes, increased exposure to this content in national avenues has the potential to exert a significant effect due to the high utilization rate of these resources. Although we cannot be certain that the small differences between our groups are large enough to be clinically relevant and practice changing, an increase in SOC images may possibly impact the dermatologic diagnostic ability of medical students, and at the minimum, increase exposure.

## 5 LIMITATIONS

The limitations of our study include a single‐institutional setting. Subgroup analyses involving students who identify as POC are slightly underpowered due to a limited sample size. Lastly, generalizability of our conclusions is limited by small outcome differences in group comparison and an overall poor performance on the survey.

## 6 CONCLUSION

Overall, students performed similarly on diagnosing dermatological diagnoses on SOC images versus White skin images. Second‐year students were more likely to correctly identify dermatological diseases overall and on White skin compared to first‐year students. There was a clear desire for more exposure to SOC dermatologic conditions.

## CONFLICT OF INTEREST STATEMENT

All authors declare that they have no conflicts of interest.

## AUTHOR CONTRIBUTIONS

**Adina Greene**: Conceptualization (lead); data curation (lead); formal analysis (supporting); investigation (lead); methodology (lead); writing – original draft (lead); writing – review & editing (lead). **Tara Ghalambor**: Conceptualization (supporting); data curation (supporting); methodology (supporting); project administration (lead); visualization (supporting); writing – original draft (supporting). **Scott Penner**: Conceptualization (supporting); data curation (supporting); formal analysis (supporting); project administration (supporting); writing – original draft (supporting). **Chase Irwin**: Conceptualization (supporting); data curation (lead); formal analysis (lead); methodology (supporting); writing – original draft (supporting); writing – review & editing (supporting). **K. Taraszka Hastings**: Conceptualization (supporting); project administration (lead); supervision (lead); writing – original draft (supporting); writing – review & editing (lead).

## ETHICS STATEMENT

Reviewed and approved by University of Arizona IRB Review Board for STUDY00001214.

## PATIENT CONSENT

Not applicable. Consent from participants was stated to be implied based on the completion of the survey.

## Data Availability

The data underlying this article will be shared with the corresponding author on reasonable request.
